# Testing the Uniformity of Surface Resistance on Large-Format Transparent Heating Glass

**DOI:** 10.3390/s23218738

**Published:** 2023-10-26

**Authors:** Stanisław Flaga, Ireneusz Dominik, Marek Szybiński

**Affiliations:** 1 Faculty of Mechanical Engineering and Robotics, AGH University of Krakow, Mickiewicza 30, 30-059 Krakow, Poland; dominik@agh.edu.pl; 2 Vitroform sp. Z.o.o., Cichawa 152, 32-420 Gdów, Poland; marek.szybinski@vitroform.pl

**Keywords:** surface resistance, transparent electrode coating, transparent heater

## Abstract

The design of a glazing package containing heating glass can make a window a radiator simultaneously. For such bulky glass to act as an effective radiator simultaneously, it should be possible to provide a constant temperature over the entire surface. The continuous surface temperature of the glass depends on the uniformity of the surface resistance of the resistive layer. This paper will demonstrate the testing of heating glass parameters using a specialised apparatus. The research will mainly focus on measuring the value and distribution of the surface resistance of the transparent heating layer. A thermographic study will verify the results. As the heating glass will be subjected to a toughening process, the effect of the toughening process parameters on the degradation of the transparent heating film will be investigated.

## 1. Introduction

**Figure 1:**
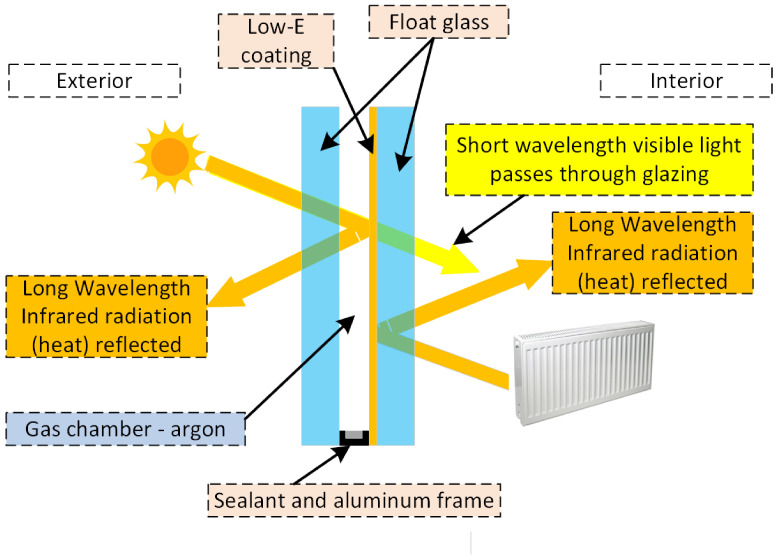
Construction of a double-glazed, single-chamber package with low-e coating.

The main properties of glass are high light transmission, relatively low thermal conductivity coefficient: 0.8 ÷ 1.2 $W/(m^{2}\cdot K)$, and good dielectric properties: 1010 ÷ 1014 (Ω· m), hard surface: 6, 7 on the Mohs scale—resistance to most chemicals [1]. From January 2021, new thermal transmittance values for architectural elements have been defined according to European Directive 2010/31/EU [2]. According to [2], glazing packages in windows must have a thermal transmittance of 0.5 or 0.6 $W/(m^{2}\cdot K)$. Such parameters are mainly possible in triple-glazed, double-chambered packages with argon-filled chambers. In experimental solutions, quadruple-glazed packages with krypton-filled chambers appear with thermal transmittance values close to 0.3 $W/(m^{2}\cdot K)$. Multi-pane packages naturally have a higher weight, requiring reinforcement of the window frames and the entire structure. In addition, they make installation more difficult due to their increased weight. Another direction to reduce thermal transmittance without increasing the weight of glazing packages is experimental double-glazing packages with a vacuum chamber with a thermal transmittance of approximately 0.47 $W/(m^{2}\cdot K)$. A promising direction for obtaining lighter glazing packages is using low-emissivity glazing [3]. Low-emissivity glazing is achieved by applying a transparent thin layer that transmits visible light and thermal radiation from the sun inwards and reflects long-wave radiation generated inside the house (Figure 1) [4].

**Figure 2:**
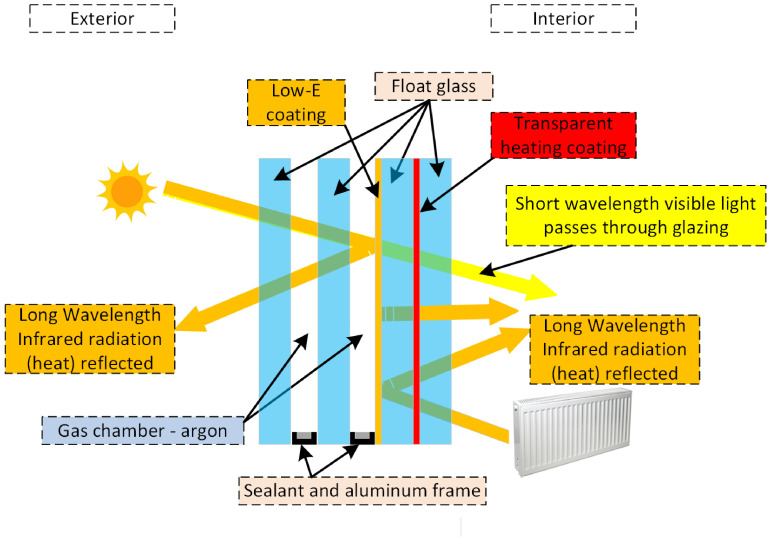
Construction of a triple-glazed, double-chambered package with a low emissivity coating and a transparent heating coating.

Reducing permissible thermal transmittance coefficients for new developments offers the possibility of reducing the heat transfer surface in radiators. The possibility of providing thermal comfort with a reduced heat transfer surface makes it possible to use the modern solution of heating glass [5]. Heating glass is obtained by applying a transparent resistive layer on one sheet of glass. Skillfully energising the resistive layer causes it to heat up and give the glass its heating property (Figure 2).

Double-glazed windows are designed with an even more complex structure that allows waste heat to be utilised [6]. Such glass is marketed under the name eGlass. eGlass is a good material for use in the glazing packages used to construct windows. A window constructed in this way is energy active—the exchange model for active windows is described in [7]. Glass is an increasingly common architectural material used to construct partition walls. Thanks to the heating coatings, a wall constructed in this way acquires the additional functionality of a radiator [8].

Applying transparent functional layers to glazing used in construction offers great possibilities [9]. Window panes can be fitted with sensory systems quite easily. In heating applications, it is possible to implement temperature sensors—such as resistance temperature detector (RTD)—on the glass [10]. One of the more recognisable functional glass solutions is the manufacture of glass-polymer-dispersed liquid crystal (PDLC) laminates. PLDC films allow transparency to be changed due to an applied voltage—automatically darkening/brightening the window [11]. Such functionality can be achieved with a layer of, for example, vanadium (IV) oxide $VO_{2}$. Transparent functional layers are the basis for creating intelligent windows [12], including those that can generate electricity [13].

The history of Transparent Electrode Coating (TEC) dates back to the 1980s. Chronologically, the first transparent TCOs (Transparent Conducting Oxides) with various additives were used. Indium tin oxide (ITO) has excellent properties, high transparency and low resistance. Its fabrication is possible using several techniques [14]. The biggest disadvantage is the increasing price of Indium. Fluorine-doped zinc oxide (FTO) [15] or aluminium (AZO) layers [16] prove to be cheaper to produce. Subsequently, carbon nanotube networks [17,18], graphene [19], metal nanowires [20], and hybrid combinations of the above and metal lattices [21] have been experimented with. Despite the multivariate approaches to creating transparent heating layers, the most cost-effective layer remains metal oxide [22]. Nowadays, heating glass is often found in refrigerated display cases with transparent doors. The heating layer is used to defrost the door. Criteria for selecting transparent conductive coatings for heating, among other things, were already presented in 2000 [23].

## 2. Methods for Measuring the Surface Resistance of Thin Conductive Films

The most important parameters characterising the electrical properties of the layers under investigation include resistivity and surface resistance $R_{s}$ electrical permeability, magnetic permeability, and dielectric loss factor [24]. $R_{s}$ will be investigated in the preliminary analysis of raw and hardened heating glasses.

### 2.1. Principle of Surface Resistance Measurement by Contact Method

**Figure 3:**
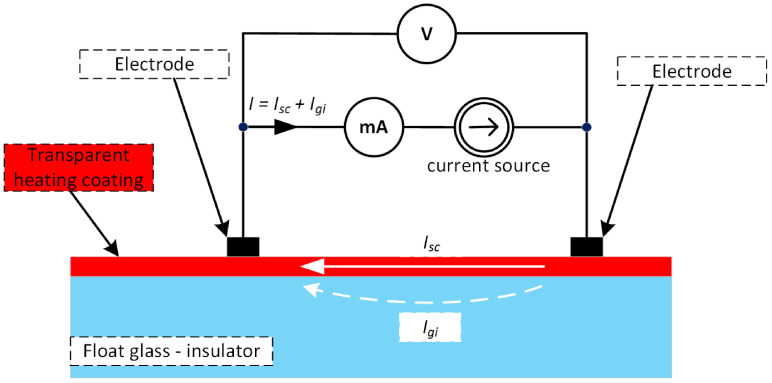
Block diagram of a two-point technical conductive layer resistance measurement.

In the case of conductive layers deposited on glass (insulated substrate), the resistance measurement is related to the part where the cross current flows through a known cross-section of the layer (square). The contribution of the current $I_{gi}$ flowing through the glass on which the conductive layer is deposited is negligibly small compared to the total measurement current in which the main contribution is the current flowing through the conductive layer $I_{sc}$—see Figure 3.

The surface resistivity $\rho_{s}\left(V\cdot m/A\right)$ is the quotient of the electrical voltage U(V) and the linear current density $J_{s}\;\left(A/m\right)$ in the conductive layer Equation (1).

(1) $$\rho_{s}=\frac{U}{J_{s}}$$

**Figure 4:**
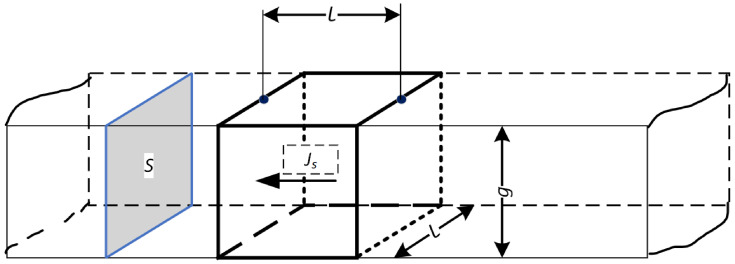
Imaging of a conductive layer in a cuboidal shape.

Figure 4 shows a conductive layer in the shape of a cuboid. The two sides of the cuboid are equal *l*.

The concept of resistance related to the square area of the layer at its conventionally assumed thickness g<<l is the surface resistance often denoted as $R_{s}$ Equation (2). Surface resistance is related to the square of the area, and its unit is $\Omega {/}_{□}$ (Ω per square)

(2) $$R_{s}=\frac{\rho_{s}}{g}$$

**Figure 5:**
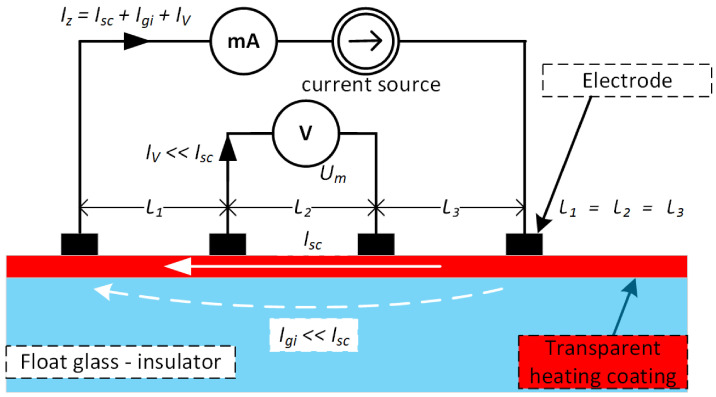
Principle of surface resistance measurement using the four-point method.

The term surface resistance $R_{s}$ describes a material property resulting in the magnitude of the current flowing in the boundary layer formed between the test material and the environment, related to a conventional surface determined by setting up the measuring electrodes (square or ring). In the planned study, it will be related to a square. The following methods of measuring surface resistance and equivalent resistance between electrodes are typical of those used in laboratory and industrial technology [25]. The most common ways of measuring the surface resistance of conductive layers are contact (non-destructive) methods which include:

- two-point method—problem with accuracy and repeatability due to lack of compensation at the thin film-electrode interface. Low-cost implementation because a simple ohmmeter can be used as the measuring instrument—here, the resistance between the two electrodes can be measured,
- four-point method [26]—accurate method-measuring instruments are specialised Figure 5—here results are often converted to $\Omega {/}_{□}$. This method is not new [27,28], but is still widely used in many fields. For example, in geophysics, to measure the shear strength of peat soil [29] or construction [30]. The exciting results of using this method to measure the in situ change in electrical resistivity during plastic deformation to characterise the deformation of metals were shown in [31].

For a layer whose thickness g is many times smaller than the distance between the measuring probe electrodes $\left(l_{1}=l_{2}=l_{3}\right)$, the surface resistance in the four-point method can be calculated from relation (3):

(3) $$R_{s}=\frac{\pi g}{ln2}\cdot \frac{U_{m}}{I_{z}}$$

### 2.2. Limitations of the Contact Method

**Figure 6:**
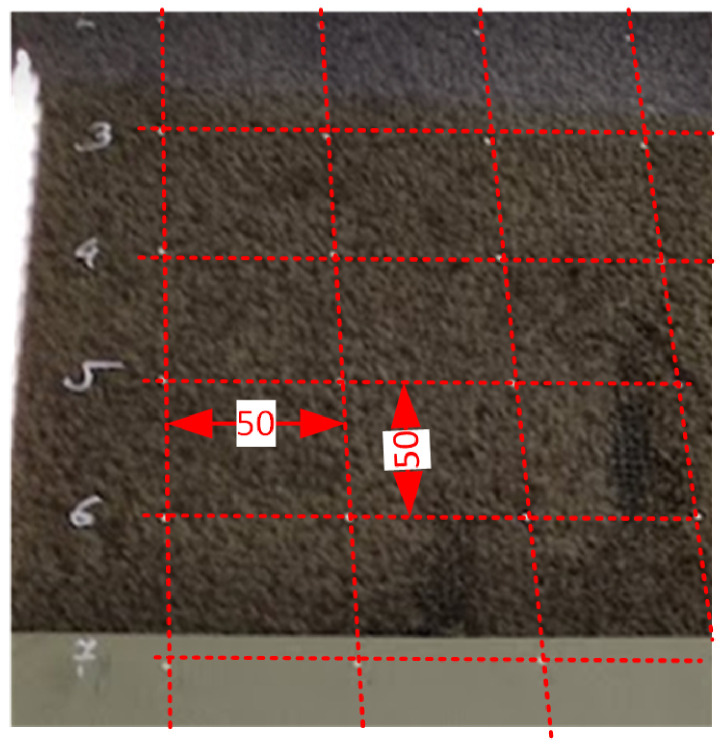
Preparation of the contact points on the glass with protective insulation layer—glass with marked points.

Contact methods can only be used for conductive layers to which we have access with measurement probes. This method cannot be used if a protective layer protects the conductive layer. Contact between the electrodes and the conductive layer cannot then be ensured. Technologically, access to the conductive layer on the coated glass with a protective layer is achieved in two steps. The first step involves applying AG 7500-88 conductive paste electrodes to the protective layer. In the second step, the coated glass with the painted electrodes undergoes quenching. During quenching, the conductive paint fuses with the conductive layer on the glass [32]. Problems arose with glass on which a conductive layer was applied, covered by an insulating protective layer. The protective layer is an insulator that prevents the measuring head from contacting the conductive layer. An attempt was made to design and build an instrument for measuring surface resistance using the four-point technical method. Conductive contact points were prepared on the heating glass in a 50 × 50 mm grid and, after hardening, were used as the basis for measurements by the four-point method and tests of the two-point method were carried out. Unfortunately, the effect of the applied contact points on the measurement results was so significant that this concept was abandoned. It could have worked better for either the two-point or four-point method. The contact points for the electrodes applied to the actual glass are shown in the Figure 6.

### 2.3. Mechanism of Operation of the Eddy Current Non-Contact Method

The eddy current phenomenon has many adverse effects in systems with magnetic circuits. Nevertheless, the eddy current phenomenon has been used successfully in defectoscopy [33] and non-contact measurements of the properties of conductive materials [34]. The magnetic interaction of an AC-powered coil achieves the excitation of eddy currents in a conductive layer. The excited eddy current flow generates its magnetic field, which interacts with the magnetic field of the excitation circuit and/or the measuring coil. In simple terms, the material property under test is a function of the measured magnetic field interaction from the eddy current on the leading circuit. It changes the impedance of the inductor. Another way to do this is to find the relationship between the material property and the voltage characteristics generated in a separate measuring coil.

## 3. Experimental Determination of the Surface Resistance of Coated Glass

**Figure 7:**
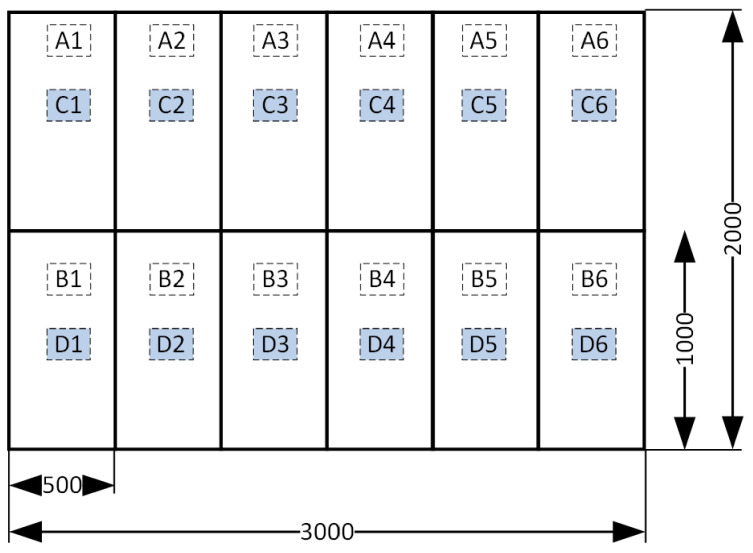
Method of extracting samples from 2 panes of TEC glass and 2 panes of NrG glass; pan 1: A1.. A6, B1..B6; pan 2: C1..C6, D1..D6.

The experiment began with sample preparation. In a synthetic summary, it can be presented as follows. Two sheets each of TEC manufactured by Pilkington (Lathom, UK) and Energy Glass (Cantu, Italy) (NrG or nRG—Industry abbreviation for glass company Guardian Glass ) measuring 3000 × 2000 mm were cut into 1000 × 500 mm samples (Figure 7). This yielded 24 samples of TEC coated glass and 24 samples of NrG glass.

All 48 TEC glass samples were tested using an M-3 instrument from Suzhou Jingge Electronics with a four-point measuring head. The NrG glass samples were tested with a SURAGUS EddyCus TF portable 1010 instrument using the eddy current phenomenon. The instrument parameters are shown in Table 1.

### 3.1. M-3 Instrument Tests

**Figure 8:**
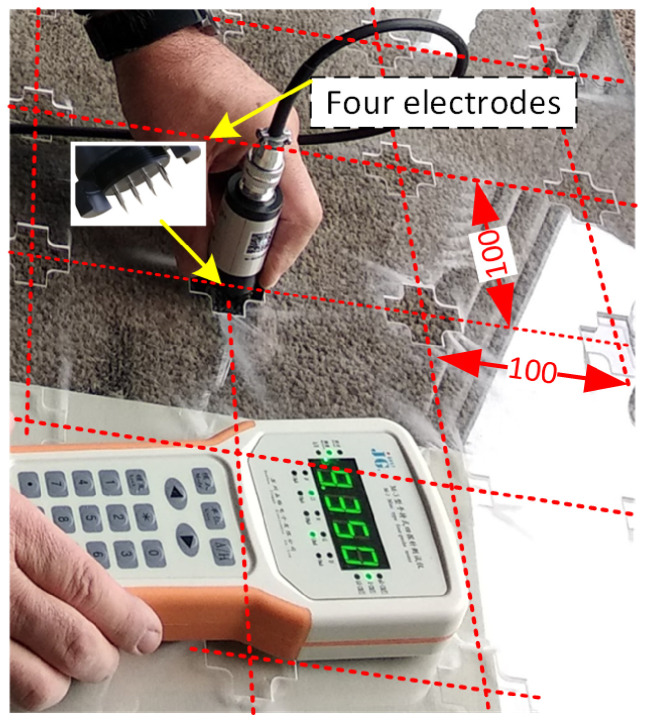
Measurement of surface resistance using the four-point method with the use of a template.

A disadvantage of contact method measurements is ensuring correct contact between the layer and the electrodes of the measuring instrument. The M-3 gauge used in this study has a head with interchangeable electrodes. It is advisable to check the correct choice of electrodes for the device by making repeated measurements at the exact locations. After an initial selection, electrodes were chosen as shown in Figure 8.

Before measuring the samples, tests were conducted on the instrument fitted with the selected electrodes. The test consisted of measuring the surface resistance of the glass several times, which, according to the manufacturer, was characterised by $Rs=9.5\;\Omega {/}_{□}$.

Measurements were taken over the next four days. Stretching the measurement over time was intended to check the instrument’s repeatability.

A 500 × 500 mm sample was used. A line was drawn connecting two opposite sides of the specimen and points at coordinates 0, 80, 160, 240, 320, and 400 mm were marked. A point 50 mm from the edge of the specimen was taken as the origin of the axis. The distance of 50 mm from the edge of the sample is due to the laser cut-off of the outer 25 mm wide frame. The collected measurements are shown in Table 2. The last two columns give the statistical parameters for the point. The Ho hypothesis for all measurements at 6 points is that the population mean equals the sample reference value of $9.5\;\Omega {/}_{□}$. The arithmetic mean is 9.55, and the Student’s *t*-test result T=1.18. The two-sided confidence interval (critical importance) read from the tables for n−1=23 degrees of freedom and significance level α=0.05is2.069. We can accept the $H_{o}$ hypothesis since the critical value is greater than the test value. This means that the measuring electrodes were selected correctly.

### 3.2. Surface Resistance Measurements of Samples

To facilitate the measurement of 48 samples with the M-3 instrument, a particular template was prepared to stabilise the measuring head of the four-point device in two directions. The slots for the measuring head were made based on a 100 mm square mesh. The template allowed the measuring head to be manually positioned in 50 positions, in two directions for each position—Figure 8. The head coordinates in the tables indicate the grid nodes in which the measurement was taken. The grid nodes are 100 mm apart in both directions. The measurement in two directions is due to the linear arrangement of the measuring electrodes.

The surface resistance of each of the 24 TEC glass samples was measured in two perpendicular directions.

### 3.3. Hardening of Heated Glass

In industrial settings, coated glass is subjected to a toughening process, sometimes toughening combined with bending. The toughening process has a destructive effect on the coatings applied to the glass. Choosing the right temperature and quenching time maintains the uniformity of the coating’s surface resistance. Selected specimens were subjected to power-path application processes and partitioning by laser evaporation of the conductive layer into heating zones. The samples summarised were subjected to the quenching process. The hardening parameters (Table 3) are based on the technologist’s experience operating the process.

## 4. Results and Discussion

A comparison of the measurements in the two directions of sample A1 of TEC glass 10 (according to the manufacturer $Rs<11.9\;\Omega {/}_{□}$ is shown in Table 4.

Statistical analysis showed that the head’s positioning did not differ from the measurements obtained. For the positioning of the head along the long side of the specimen, a mean value of 9.21 was obtained with σ=0.21, and for the positioning along the shorter side, a mean value of 9.19 was obtained with σ=0.171. The normality of the distribution of the results obtained will confirm the homogeneity of the surface resistance of the tested layers. One of the more commonly used tests for normality of distribution is the Shapiro–Wilk test. It has its weaknesses. It can be overly sensitive for large samples, but this can be seen as an advantage in this case. For the calculations, Statistica software was used in the version with the sample size limitation for the Shapiro–Wilk test of 2000. In the analyses described here, the sample size for individual samples of heating panes A1..A6, B1..B6 was 50. For the entire 3000 × 2000 mm panel, the test involved 50 × 12 = 600 samples. The data for both head arrangements were subjected to the Shapiro–Wilk test for normality of distribution and received at the 0.05 significance level *p* parameter values of, respectively, 0.469 and 0.051. Such results entitle us to accept the hypothesis of an $R_{s}$ normal distribution—similar mean values and small σ show that $R_{s}$ is the same in both directions. Here, the results of one sample are shown, but similar results were obtained for the other samples. The normality of the $R_{s}$ distribution was tested at a significance level of 0.05 for the whole panels using all AB and CD samples. A p=0.0672 was obtained for the AB samples, and a p=0.0712 was calculated for the CD samples. This is essential for the designer because it allows heating elements to be cut from a large-format glass sheet in any direction.

The nRG glass with the eddy current instrument was measured with a single alignment of the measuring head. This is due to the instrument’s design, which averages the measurement over a circular area with a diameter of 80 mm. As a result of the averaging, very similar results were obtained over the entire sheet. An average of 9.31 was obtained for the whole sheet at σ=0.011.

After the quenching process, the homogeneity of the surface resistance of the quenched samples was examined again. Table 5 summarises the measurement results of sample B2 before and after the quenching process. The mean value of $R_{s}$ after the quenching process increased from $9.31\;to\;9.43\;\Omega {/}_{□}$), which is a result of the oxidation of the coating. Tests for normality of the $R_{s}$ distributions showed that, at a significance level of 0.5, the sample before quenching (p=0.069) and after quenching (p=0.117) retains homogeneity of the resistive surface.

By observing the other samples, it can be concluded that the optimum quenching temperature is 675 ${}^{\circ }$C with a time below 380 s. What happens to the resistance coating when the quenching parameters exceed the permissible values is shown in sample B5 quenched at 680 ${}^{\circ }$C with a time of 320 s—Table 6.

After hardening with such parameters, results above $10\;\Omega {/}_{□}$ started to appear. The average resistance increased to $9.56\;\Omega {/}_{□}$. The Shapiro–Wilk test rejected the hypothesis of normality of the $R_{s}$ distribution on the sample after quenching $\left(p=9.22\cdot 10^{-8}\right)$.

### 4.1. The Coated Heater Concept

**Figure 9:**
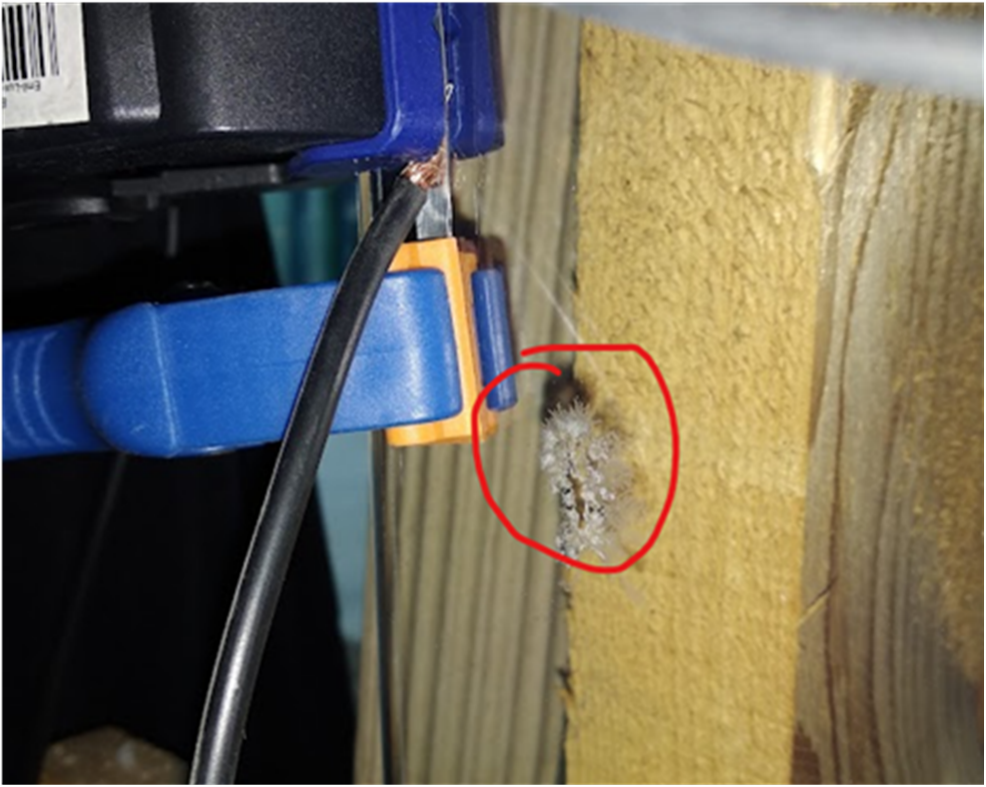
Scorching of the heating coating at point feed—directly by clamping the feed line.

Glass with an applied resistive layer behaves like a typical resistive load, which allows simple control in both DC and AC systems. There is a problem with using power on the thin film. This is due to the small thickness of the transparent film. For this reason, the supply electrodes must be in contact with the transparent layer over a large area so that the maximum current density values are not exceeded—resulting in damage to the resistive surface. The effect of exceeding the maximum in-circuit current density (here 70 kA/m${}^{2}$ in-circuit current of 7 A through a contact area of 0.0001 m${}^{2}$ ) at the contact copper wire (cable) pressed against the layer surface is shown in Figure 9. Simple contact connections are possible for large contact areas between the conductor and the conductive layer. Silver-plated intermediate electrodes can increase the contact area between the conductor and coating. Such a connection is simple but requires further research into its durability.

**Figure 10:**
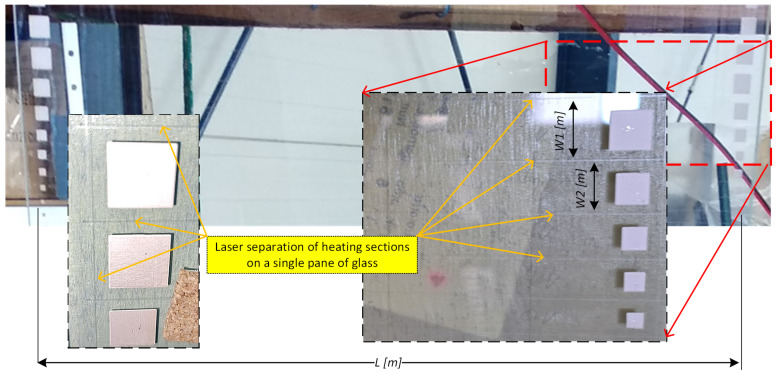
Examples of ceramic paint supply paths for laser-separated heating fields.

A typical solution is for electrodes painted with conductive paint. Paints of this type achieve the best mechanical and electrical performance after quenching. An example of electrodes made of conductive paint is shown in Figure 10.

Experimental studies show that the power density delivered to radiators Equation (4) without forced air circulation should vary between 350 ÷ 1200 W/m${}^{2}$. With a resistive load, the calculation of the supplied power is straightforward and follows directly from the Formula (5).

(4) $$P_{d}=\frac{P}{s}$$

(5) $$P=U\cdot I=\frac{U^{2}}{R}$$

where *P*—power of the heater (W); *U*—supply voltage (V); *I*—current in the heater circuit (A); *R*—resistance of the sheath between the supply electrodes (Ω); $P_{d}$—power density (W/m${}^{2}$); *s*— active area of the heater - heating layer (m${}^{2}$).

**Figure 11:**
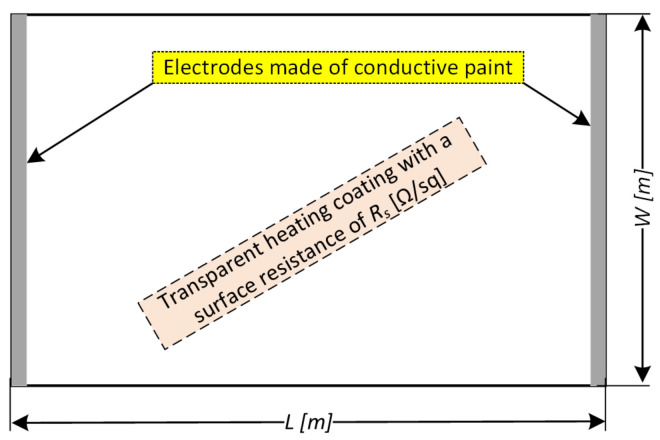
Model for calculating the resistance between two parallel electrodes supplying the heating layer.

As previously mentioned, the characteristic feature of the heating layers is the surface resistance expressed in $\Omega {/}_{□}$. Having information on the surface resistance Rs, the designer should plan the surface of the heater to obtain the assumed power density at the known parameters of the power source. For this purpose, the resistance between the supply electrodes should be determined. In the most common case—parallel electrodes (Figure 11)—the resistance R is calculated from the Formula (6):

(6) $$R=\frac{R_{s}\cdot L}{W}$$

At the design stage of the radiator system, the designer can choose from an assortment of available glass panes with heating coatings and influence the power density not only by the parameters of the power source but also by the dimensions of the active part of the glass pane. A single pane of glass does not necessarily constitute a single heater. With a laser torch within a single pane of glass, multiple heating zones can be separated by vapourising the coating without disturbing the structure of the glass. What such separation looks like on the glass for optimising electrode dimensions is shown in Figure 10. The cross-section of the printed paths from the ceramic conductive ink was experimentally modelled. A 500 × 1000 mm format was prepared on which the conductive layer was divided into rectangular strips of widths: 2, 3, 4, 5, 6, 8, 10, 12, 12, 10, 8, 6, 5, 4, 3, 2 mm and this sequence was repeated five times on the Figure 10 format. The dimensions of the strips and electrodes were calculated for a power density of approximately 400 W/m${}^{2}$ at 30 V.

Each strip had electrodes painted with ceramic conductive paint. The electrodes were square-shaped with a side proportional to the width of the strip. After hardening, the resistance of the strips was tested using the two-point method and thermography. The latter was chosen for its intrinsic ability to study heat transfer phenomena [35,36,37,38]. Only the width of the painted electrodes was analysed. The electrodes are applied by screen-printing, and their thickness is due to this technology. It has been experimentally verified that painting successive layers of electrodes (e.g., two or more layers, one on top of the other) has no effect and that the multilayer electrodes degrade quickly. This is understandable because, in this case, the contact surface of the conductive paint with the heating layer is essential, not the thickness of the electrode itself. The thermographic measurements show that the optimum widths of the conductive paths for the respective power densities are 6 mm for 250 W/m${}^{2}$, 8 mm for 500 W/m${}^{2}$ and 10 mm for the range up to 2 kW/m${}^{2}$. Higher power densities were not analysed because they are beyond the scope of application in heating windows that are architectural elements.

### 4.2. Verification of Uniformity of Temperature Distribution by Thermography

To verify the thesis that a uniform distribution of $R_{s}$ on the surface of the coating glass will result in a uniform temperature distribution, thermographic tests were carried out using a HIKMICRO G40 camera. The most important parameters of the HIKMICRO G40 camera are summarised in Table 7.

**Figure 12:**
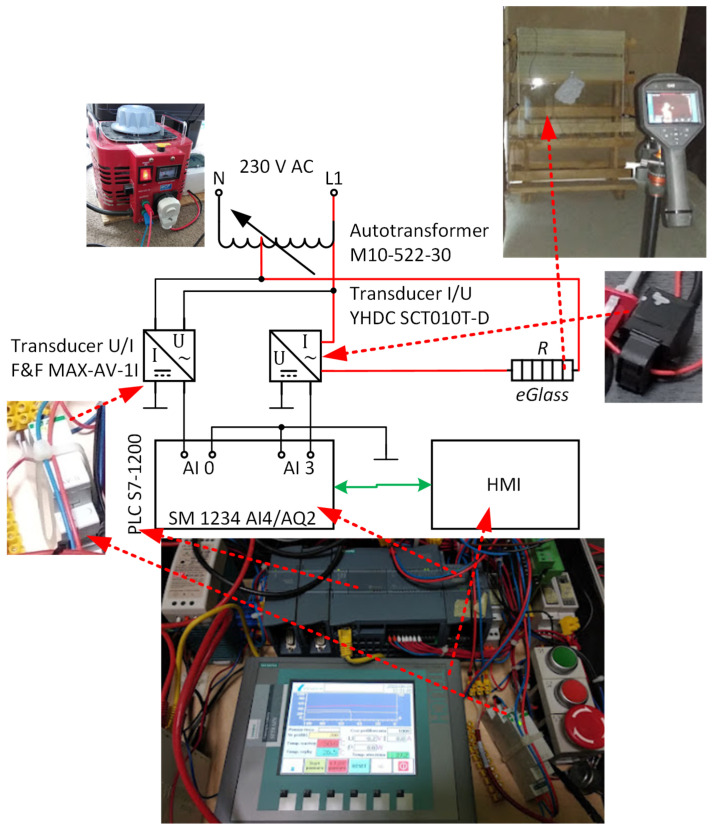
Measurement diagram of the heating pane supply parameters for thermographic tests.

The heating zone electrodes were supplied with AC from an autotransformer, which facilitated changing the power supply parameters (Figure 12).

**Figure 13:**
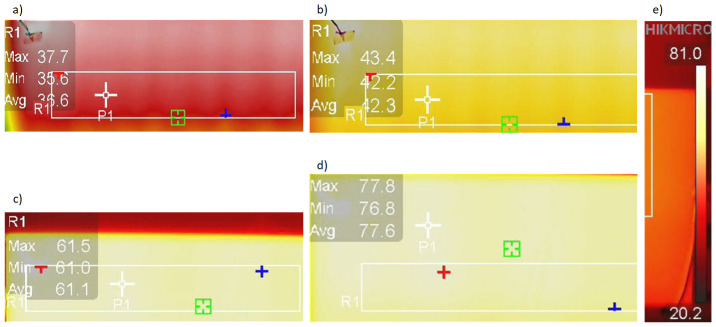
Thermograpic images were taken when the radiator was heated for temperatures: (**a**) 36.6 ${}^{\circ }$C, (**b**) 42.3 ${}^{\circ }$C, (**c**) 61.1 ${}^{\circ }$C, (**d**) 77.6 ${}^{\circ }$C, (**e**) false color temperature bar.

The measurement system uses an S7-1200 controller, which will ultimately be used to implement advanced control algorithms for the heating glass. Two 400 × 450 mm heating zones were separated on specimen B2 before toughening. The electrodes supplying the heating zones were made of conductive paint applied before quenching. The electrode dimensions are 400 × 10 mm. The actual resistance between the two parallel electrodes was 8.55 Ω. The calculated resistance value for $R_{s}=9.56\;\Omega {/}_{□}$ was 8.49 Ω. The thermographic camera was 3 m away from the sample. The study started with determining the reflected temperature using a Lambert heat sink. The measurement field was delineated by a rectangle which covered a section of the heating zone measuring 300 × 100 mm. Thermographic images were taken when the radiator was heated for average temperatures in the measuring field equal to 36.6, 42.3, 61.1 and 77.6 °C. The values of the average temperatures are not ’round’ because the heating took place in a system without control. As shown in Figure 13, the differences between the minimum and maximum temperatures are minor, which allows us to assume that achieving a uniform temperature distribution on the glass surface with a uniform $R_{s}$ is possible.

## 5. Conclusions

Heating glass, although known for many years, is now being rediscovered. It is the result of the progress of material technology, which allowed the creation of thin and transparent layers with surface resistance that give the designer a lot of freedom in shaping the geometry of the radiator. A thorough examination of the homogeneity of selected heating layers allows for the creation of transparent heaters with an even temperature distribution over the entire heated surface. The section on $R_{s}$ measurements showed that both TEC and nRG large-format coated glass panes maintain the uniformity of the surface resistance distribution. This is essential for designers of glass radiators because it does not matter from which part of the pane their design will be cut. Tempering of the coating glass is necessary due to its properties and to obtain the proper condition of conductive paints used as electrodes. It was shown that the appropriate selection of tempering parameters slightly affects the average resistance value–the surface resistance of the coating glass after toughening increases slightly but does not affect the uniformity of the $R_{s}$ distribution. This paper shows that testing the normality of the $R_{s}$ distribution (here, Shapiro–Wilk tests) is sufficient to conclude that the $R_{s}$ distribution is homogeneous. Thermographic tests have experimentally proven this, which shows that a uniform temperature distribution was obtained on the glass after tempering. The ease of separating transparent heating zones on large glass surfaces sets further directions of research towards intelligent and ecological control of many distributed heaters.

## Figures and Tables

**Table 1:** The parameters of the instruments used for measuring surface resistance.

Instrument	Suzhou Jingge Electronics M-3	SURAGUS EddyCus TF Portable 1010
**Measurement head**	ST2258B-F01 thin film square resistance linear four-point probe	integrated
**Measurement technology**	4-point probe	Eddy current sensor
**Measurement mode**	contact	Realtime at constant distance or contact
**Measurement spot**	Straight line 4 mm	Diameter 40 mm
**Sheet resistance range**	2 ÷22 $\;\Omega {/}_{□}$	0.001 ÷100 $\Omega {/}_{□}$
**Accuracy**	±1%FSB ± 2LSB (Full Scale Range, Least Significant Bit)	0.001 ÷ 50 $\Omega {/}_{□}$: <3% 50 ÷ 100 $\Omega {/}_{□}$: <5%

**Table 2:** Four-fold measurement of the surface resistance of the same points with the M-3 instrument.

Measurement Point No.	Position	Surface Resistance [$\Omega {/}_{□}$]				Average	Standard Deviation
	**[mm]**	**Day 1**	**Day 2**	**Day 3**	**Day 4**	[$\Omega {/}_{□}$]	
1	0	9.47	9.63	9.53	9.35	9.50	0.14
2	80	9.60	9.53	9.74	9.33	9.55	0.20
3	160	9.30	9.65	9.28	9.74	9.49	0.29
4	240	9.89	9.44	9.54	9.69	9.64	0.24
ine 5	320	9.58	9.46	9.30	9.77	9.53	0.24
6	400	9.69	9.41	9.79	9.46	9.59	0.22

**Table 3:** Hardening parameters for selected samples—TEC and NrG glasses.

Temperature (${}^{\circ }$C)	Time (s)	Sample No
670	320	C1, C6, D1
675	290	A2, A3, A5
675	320	B2, B3, B4, C2, C4, C5
675	380	D2, D4, D5
680	320	A6, B5, D6

**Table 4:** Results of the four-point method for sample A1 with two perpendicular orientations of the measuring head.

Head	Head Dir “|” ($\Omega {/}_{□}$)					Head	Head Dir “-” ($\Omega {/}_{□}$)				
**Coords**	**1**	**2**	**3**	**4**	**5**	**Coords**	**1**	**2**	**3**	**4**	**5**
1	9.29	9.60	9.19	9.33	9.16	1	9.11	9.27	8.80	9.14	9.13
2	9.15	9.52	9.29	9.37	9.34	2	9.11	8.70	9.11	9.11	9.04
3	9.21	9.34	9.41	9.07	9.29	3	9.09	8.84	9.03	9.27	9.45
4	9.23	9.04	9.40	9.50	9.36	4	9.25	9.10	9.17	9.19	9.35
5	9.33	9.17	9.07	9.05	9.12	5	9.10	9.26	9.20	9.52	9.33
6	9.20	9.13	9.04	8.91	9.34	6	9.11	9.17	9.03	9.38	9.66
7	8.91	9.75	9.19	8.92	8.89	7	9.22	9.03	9.09	9.17	9.33
8	9.17	8.97	8.99	8.86	9.53	8	9.12	9.33	9.17	9.04	9.02
9	8.91	9.55	9.68	9.25	9.26	9	9.12	8.91	9.11	8.91	9.14
10	9.05	8.82	9.12	9.02	9.11	10	9.16	9.06	9.00	9.33	9.30

**Table 5:** Summary of $R_{s}$ measurements of sample B2 before and after the hardening process.

Head Coords	“B2” Surface Resistance ($\Omega {/}_{□}$) *before Hardening*					Head Coords	“B2” Surface Resistance ($\Omega {/}_{□}$) ***after Hardening: 675 ${}^{\circ }C$, 320 *s****				
	**1**	**2**	**3**	**4**	**5**		**1**	**2**	**3**	**4**	**5**
1	9.38	9.72	9.03	9.10	9.08	1	9.40	9.49	9.35	9.20	9.37
2	9.38	9.56	9.34	9.37	9.14	2	9.34	9.46	9.29	9.26	9.47
3	9.02	9.53	9.43	9.50	9.42	3	9.49	9.40	9.41	9.30	9.23
4	9.35	9.51	9.56	9.36	9.33	4	9.35	9.62	9.57	9.48	9.13
5	9.05	9.33	9.23	9.30	9.38	5	9.59	9.33	9.26	9.85	9.56
6	9.30	9.17	9.27	9.35	9.22	6	9.42	9.49	9.14	9.66	9.54
7	9.33	9.41	9.51	9.06	9.34	7	9.46	9.68	9.76	9.58	9.51
8	9.19	9.50	9.40	9.29	9.35	8	9.64	9.55	9.78	9.36	9.24
9	9.17	9.45	9.23	9.16	9.35	9	9.37	9.54	9.49	9.49	9.42
10	9.27	9.03	9.01	9.55	9.33	10	9.42	9.35	9.21	9.31	9.11

**Table 6:** Summary of $R_{s}$ measurements of sample B2 before and after the hardening process.

Head Coords	“B5” Surface Resistance ($\Omega {/}_{□}$) *before Hardening*					Head Coords	“B5” Surface Resistance ($\Omega {/}_{□}$) ***after Hardening: 680 ${}^{\circ }C$, 320 *s****				
	**1**	**2**	**3**	**4**	**5**		**1**	**2**	**3**	**4**	**5**
1	9.40	9.29	9.35	9.60	9.51	1	9.30	9.13	9.40	9.66	9.27
2	9.29	9.32	9.29	9.82	9.87	2	9.50	9.59	9.40	9.59	9.72
3	9.52	9.22	9.33	9.40	9.40	3	9.29	9.30	9.34	9.30	12.03
4	9.30	9.53	9.39	9.62	9.35	4	9.57	8.91	9.30	9.79	9.23
5	9.42	9.64	9.46	9.79	9.74	5	9.40	9.19	9.32	9.41	9.40
6	9.62	9.33	9.35	9.55	9.51	6	9.44	9.65	9.14	9.29	8.68
7	9.38	9.56	9.40	9.65	9.29	7	9.48	9.24	10.36	9.44	11.33
8	9.23	9.21	9.56	9.47	9.55	8	9.25	9.15	10.26	10.75	9.19
9	9.47	9.41	9.56	9.45	9.58	9	10.16	9.15	10.59	10.04	9.78
10	9.40	9.39	9.27	9.42	9.52	10	9.15	9.30	9.17	9.29	9.46

**Table 7:** Thermal camera specifications.

Parameter	Value
Thermal resolution	480 × 360 (172,800 pixels)
Noise Equivalent Temperature Difference (NETD)	<35 mK (@ 25 ${}^{\circ }$C, F# = 1.0)
Temperature measurement range	−20 ${}^{\circ }$C to 650 ${}^{\circ }$C
Accuracy	±2 ${}^{\circ }$C/3.6 ${}^{\circ }$F, ±2%
Spectral Range	8–14 µm
Instantaneous Field of View (IFOV)	0.68 mrad
